# Subcutaneous emphysema and pneumomediastinum following orbital blowout pathological fracture in a cat with nasal lymphoma: a case report

**DOI:** 10.1186/s12917-023-03722-0

**Published:** 2023-09-15

**Authors:** Ye-In Oh, Heejeong Hong, Tae-hee Kim, Kyuyong Kang, Junghee Yoon, Hwa-Young Youn, Kyoung-Won Seo

**Affiliations:** 1 Department of Veterinary Internal Medicine, College of Veterinary Medicine, Kyungpook National University, Daegu, 41566 Republic of Korea; 2 Department of Veterinary Clinical Science, College of Veterinary Medicine, Seoul National University, Seoul, 08826 Republic of Korea; 3 College of Veterinary Medicine, Research Institute for Veterinary Science, Seoul National University, Seoul, 08826 Republic of Korea

**Keywords:** Lymphoma, Cat, Chemotherapy, Blowout fracture, Pneumomediastinum

## Abstract

**Background:**

Subcutaneous emphysema and pneumomediastinum are rare complications associated with orbital blowout pathological fracture.

**Case presentation:**

A 7-year old, castrated male Abbysinian cat presented with anorexia, lethargy, nausea, eyelid swelling, nasal discharge, and sneezing. Based on the clinical and diagnostic work-up, the cat was diagnosed with T cell high-grade nasal lymphoma associated with orbital pathological fracture due to the tumour invasion. After chemotherapy, the cat showed massive subcutaneous emphysema from frontal region to abdomen and pneumomediastinum due to orbital blowout pathological fracture. As the nasal mass decreased in volume; the air had moved from the maxillary sinus to the subcutaneous region and the mediastinum through fascial planes in the head and neck region.

**Conclusions:**

This is a first case report of a massive subcutaneous emphysema and pneumomediastinum due to an orbital blowout pathological fracture following chemotherapy in feline nasal lymphoma in veterinary medicine.

## Background

An orbital blowout fracture is an isolated fracture of the orbital walls without involvement of the orbital rim [1]. In human, this is a fracture of the orbital floor and medial wall, which causes contents of the orbit to escape into the maxillary and ethmoid sinuses, resulting in enophthalmos, impaired ocular motility, diplopia [2], tissue herniation, hypoglobus [3], facial deformities, and dysfunction of the visual apparatus [1]. Physical forces can cause soft-tissue damage as well as blowout fracture of the orbit, and the resulting intraorbital edema can also affect structural deformation [1].

Subcutaneous emphysema in cats is mainly caused by endotracheal intubation, trauma, tracheal foreign body, endoscopy, and surgery [4]. Rarely, aerodigestive injuries such as esophageal or tracheal rupture may result in pneumomediastinum [5], leading to rare but life-threatening events [6]. Retropharyngeal emphysema or pneumomediastinum can occur very rarely due to facial fractures such as orbital blowout fracture, and subcutaneous emphysema is usually caused by air movement along the fascial planes to the subcutaneous area; the inverse direction is very rare [5–8]. The paths by which subcutaneous emphysema develops into pneumomediastinum can be (1) through the retropharyngeal space to the submandibular space or (2) along the vascular sheaths in the neck to induce subcutaneous emphysema and pneumothorax [9]. Although a few human cases of extensive subcutaneous emphysema and pneumomediastinum caused by orbital blowout fracture have been reported [5–7, 10], that has been not yet reported in veterinary medicine.

Herein, we report a rare case of massive subcutaneous emphysema and pneumomediastinum consequent to orbital blowout fracture after chemotherapy for nasal lymphoma as well as its clinical follow-up in a cat.

## Case presentation

**Fig. 1:**
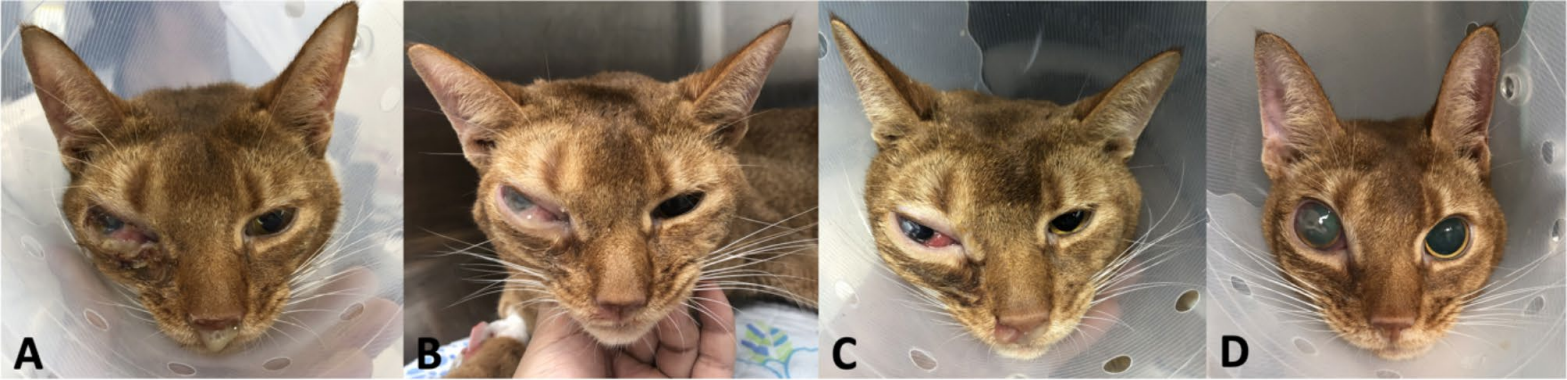
Clinical photographs showing therapeutic response of nasal lymphoma after chemotherapy. **A**) Before chemotherapy. **B**) 9 h after chemotherapy (injection of L-asparaginase 400 U/kg subcutaneously). **C**) 20 h after chemotherapy. **D**) 45 h after chemotherapy (24 h after injection of vincristine 0.5 mg/m^2^ intravenously)

**Fig. 2:**
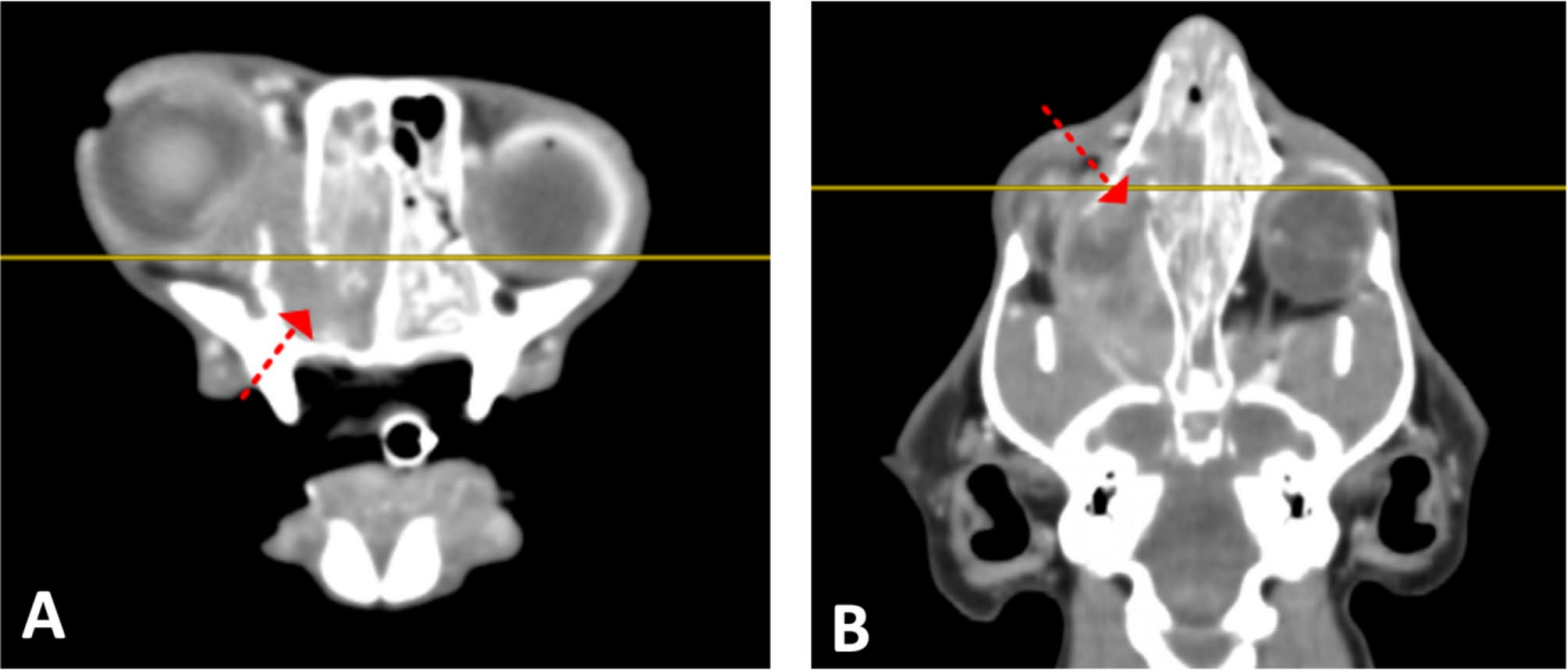
Transverse (**A**) and dorsal (**B**) plane computed tomography images after contrast enhancement. There is a mass protruding from the right nasal cavity (arrows) with lytic change of the medial wall of the right orbit. The mass is characterized by heterogeneous contrast enhancement with rim enhancement, causing deviation of the right eyeball

**Fig. 3:**
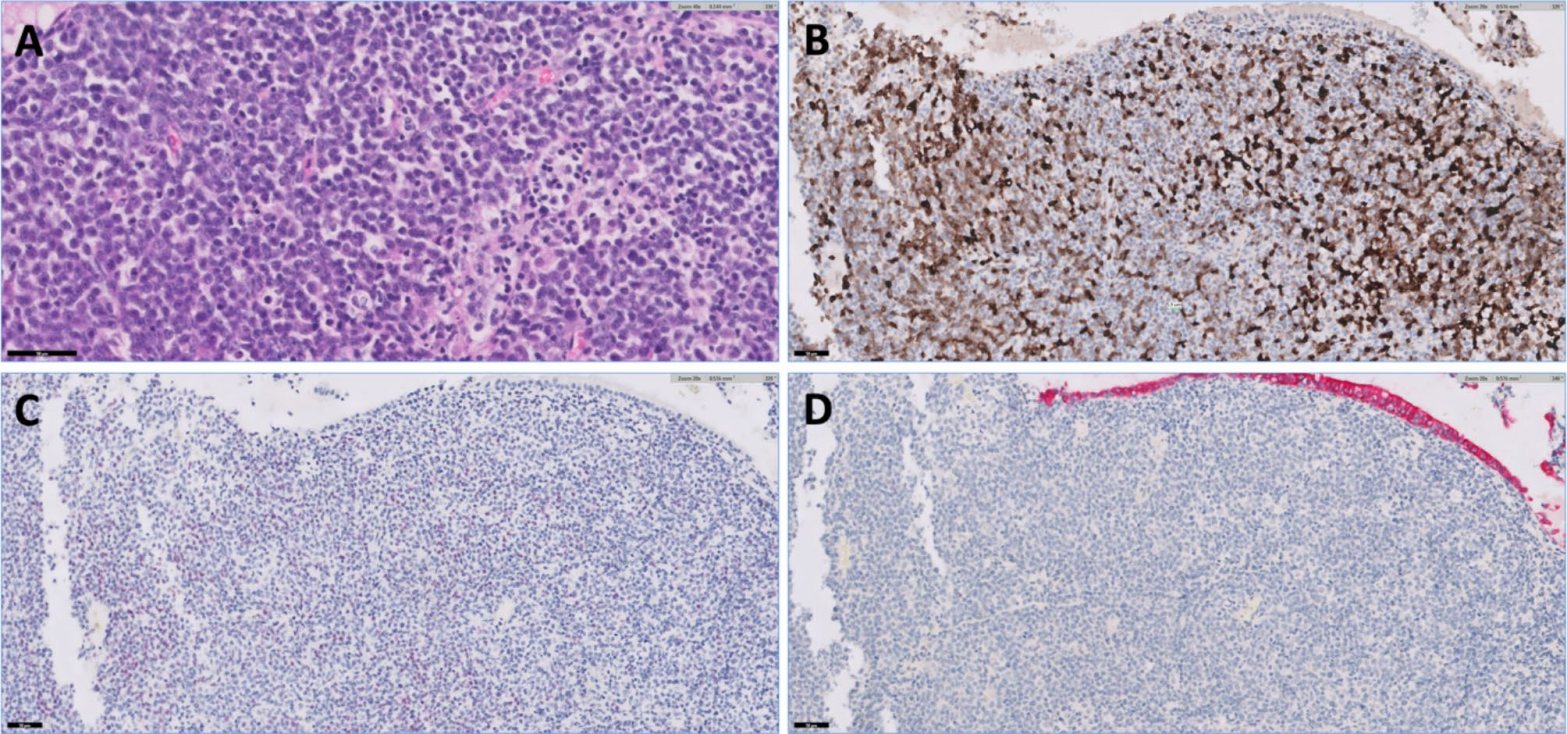
Histopathology and immunohistochemistry of the nasal mass. **A**) Histopathology (hematoxylin and eosin stain) of the nasal mass. Neoplastic cells have distinct cell borders, minimal cytoplasm, and occasionally one prominent nucleolus. There is moderate anisocytosis and anisokaryosis (**×**40). **B**) CD3 stain. Approximately 60–75% of cells demonstrate positive cytoplasmic immunoreactivity, with a variable pattern (**×**20). **C**) PAX5 stain. Approximately 25% of cells demonstrate often faint nuclear immunoreactivity. **D**) Cytokeratin AE1/3 stain. Neoplastic cells are diffusely negative. There is an internal positive control of surface respiratory epithelium

A 7-year old, castrated male Abyssinian cat presented with decreased appetite, lethargy, nausea, eyelid swelling, nasal discharge, and sneezing for 3 weeks (Day 0). On physical examination, the cat showed swelling of the right orbit and nose, bilateral purulent nasal discharge, right-sided exophthalmos and purulent ocular discharge (Fig. 1A). The patient underwent a blood analysis (complete blood count, serum-chemistry, electrolyte, feline serum amyloid A, symmetric dimethylarginine, prothrombin time, and activated partial thromboplastin time), diagnostic imaging (thorax radiograph, skull radiograph, and computed tomography [CT]), histopathology and immunohistochemistry (CD3, PAX5, and cytokeratin) of nasal cavity mass, and PCR for antigen receptor rearrangement (PARR). The specimen of mass was collected by endoscopy-guided biopsy. Blood work abnormalities included mild leukocytosis (white blood cell count 22,120/uL; reference range, 6,300 − 19,600/uL) and mild hyperglycemia (blood glucose 163 mg/dL, reference range 60–130 mg/dL). Chest x-rays revealed no abnormalities and skull x-rays revealed a round soft tissue opacity protruding into the right eye region. CT showed a nasal mass protruding from the rigth nasal cavity with lytic changes of the medial wall of the right orbit (Fig. 2). Results of histopathology, immunohistochemistry, and PARR led to a diagnosis of T cell high-grade nasal lymphoma (Fig. 3).

The cat showed anorexia on Day 14, at which point chemotherapy was started with the L-COP protocol (Fig. 1B). Chemotherapy was started with subcutaneous (s.c.) L-asparaginase at 400 U/kg. Diphenhydramine (2 mg/kg s.c.) and dexamethasone (0.5 mg/kg s.c.) were given as a pretreatment 15 min before L-asparaginase injection. The cat was treated in hospital with fluid therapy, intravenous [i.v.] tramadol (2 mg/kg twice a day), marbofloxacin (4 mg/kg s.c. once a day), prednisone (2 mg/kg twice a day), famotidine (1 mg/kg twice a day), gabapentin (10 mg/kg twice a day), and mirtazapine (1.88 mg every other day) were prescribed. Anorexia was resolved on the morning of Day 15. On Day 15, Vincristine (0.5 mg/m^2^ i.v.) and maropitant (1 mg/kg i.v., once a day) were administered, and 0.25 mg oral prucalopride per day was prescribed (Fig. 1C). On Day 16, the patient became bright and alert, and clinical symptoms of eye protrusion and nasal congestion dramatically improved (Fig. 1D) and was discharged from the hospital.

**Fig. 4:**
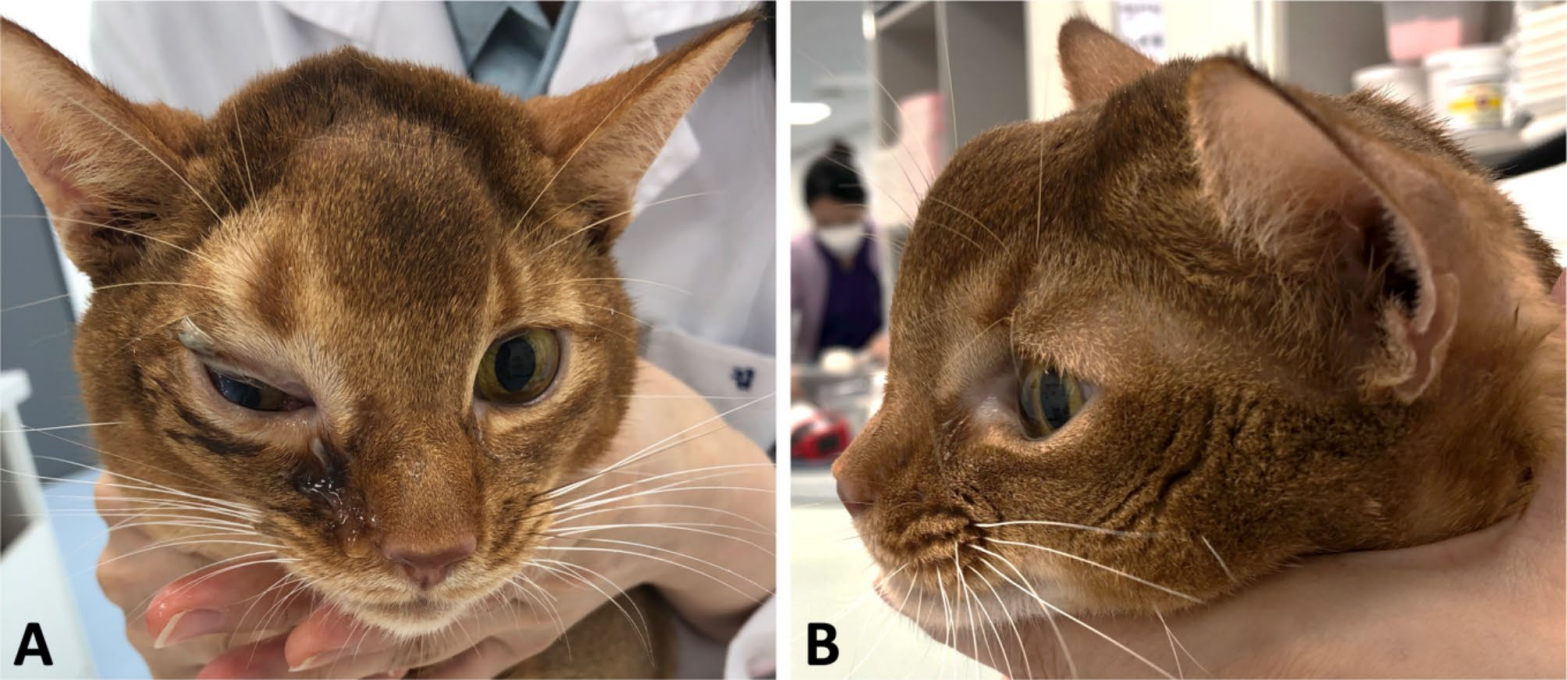
Subcutaneous emphysema of the right frontal region in a cat with nasal lymphoma after chemotherapy

**Fig. 5:**
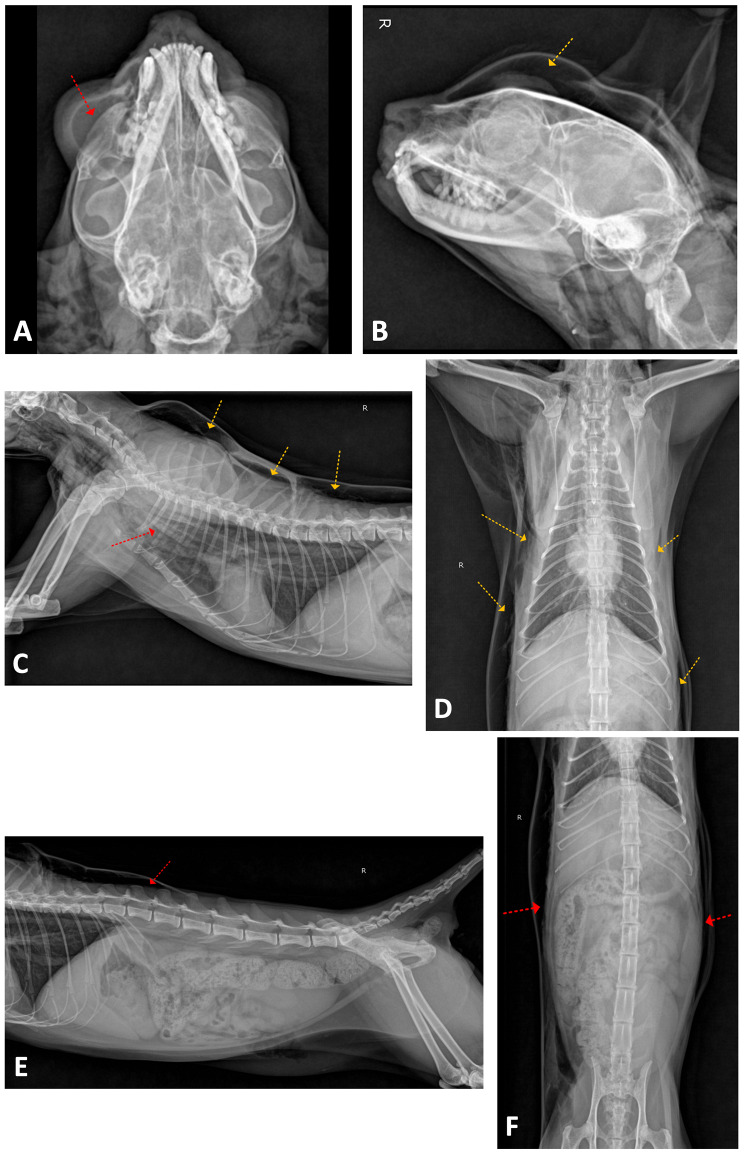
Dorsoventral (**A**) and right lateral (**B**) radiographic images of the head present lateral deviation of the right eyeball (red arrow) and subcutaneous emphysema dorsal to the skull (orange arrow). Right lateral (**C**) and ventrodorsal (**D**) radiographic images of the thorax present marked differentiation of mediastinal structures (red arrow) consistent with pneumomediastinum and multiple compartmentalized subcutaneous lucencies in overall body surfaces (orange arrows). Right lateral (**E**) and ventrodorsal (F) radiographic images of the abdomen also present subcutaneous emphysema (red arrows)

**Fig. 6:**
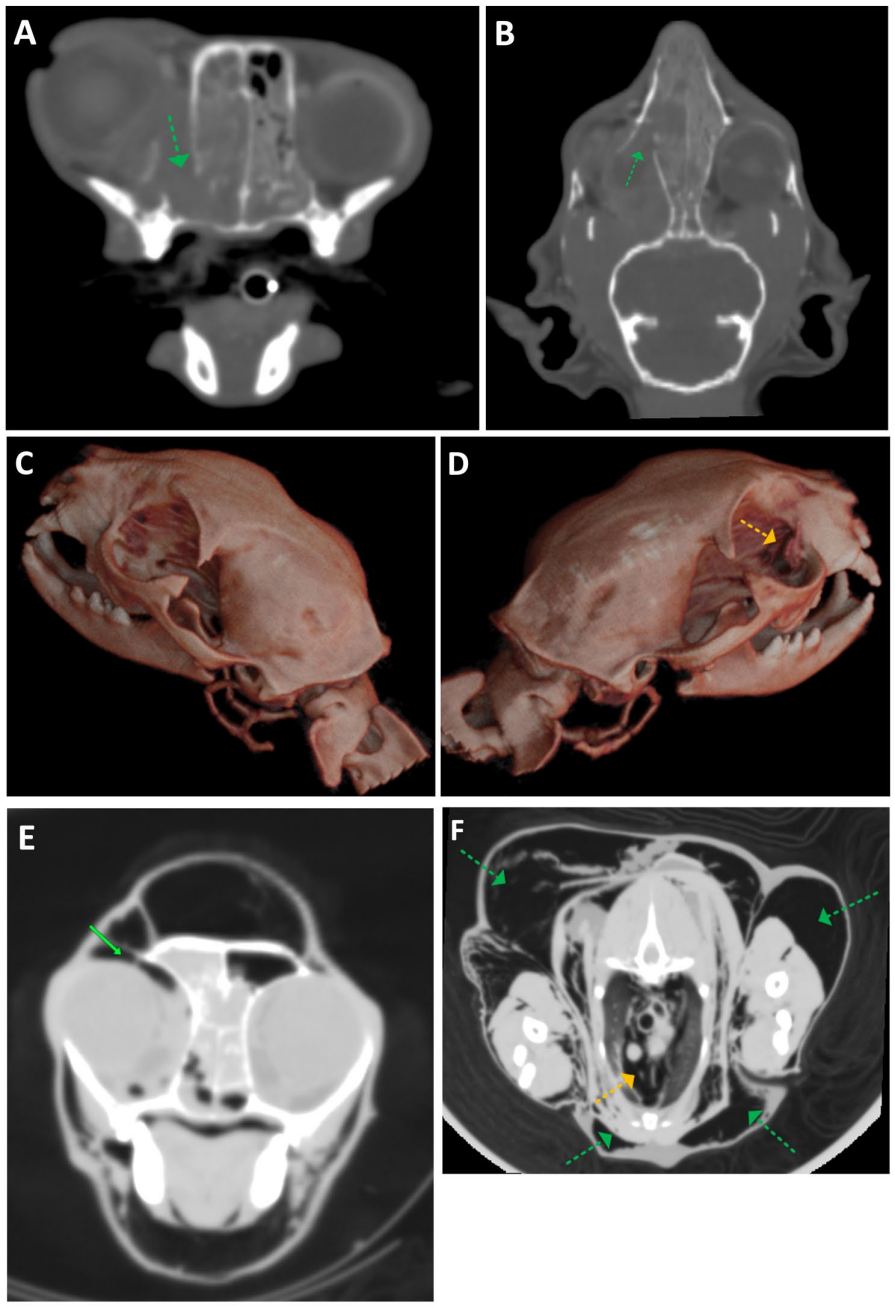
(**A**) Transverse and (**B**) dorsal plane computed tomographic images with window settings optimized for visualizing bones. The right lacrimal bone shows lytic change (arrows). (**C**) 3D volume-rendering image of the right side of the skull shows bone lysis and lateral displacement of the fragmented lacrimal bone (arrow), which can be compared to (**D**) the normal left side. (**E**) Transverse plane CT with lung mode settings shows periorbital gas (arrow) and (**F**) marked subcutaneous emphysema (green arrows) and pneumomediastinum (yellow arrow)

On day 23, the patient was still bright and alert, except for the return of right orbital edema following chemotherapy (Fig. 4). Respiration was normal on physical examination, and palpation of the swollen region revealed marked crepitus and fluctuation. There were no injuries or traumatic events. Palpable subcutaneous emphysema extended over the right side of the forehead, the scapular region, and along the abdomen. The thoracic radiographs and CT were performed which revealed massive subcutaneous emphysema from the skull to thorax region and pneumomediastinum (Figs. 5 and 6). Before the chemotherapy, the mass was filling the space around the orbital fracture site; after the chemotherapy, the size of the mass decreased dramatically, allowing the inspirated airflow to move from the nasal cavities through the orbital fracture site to the subcutaneous region and the mediastinum. Therefore, the patient developed massive subcutaneous emphysema and pneumomediastinum, with shrinkage of the nasal mass via chemotherapy allowing airflow through the previously documented orbital blowout pathological fracture.

Conservative therapy, such as whole-body bandage treatment and subcutaneous drainage using a negative-pressure syringe, was implemented to reduce air movement. The patient’s general body condition and respiratory status were normal on Day 25, and the physical examination showed an increase in the subcutaneous emphysema after bandaging. In order to continue the treatment for lymphoma, 200 mg/m^2^ cyclophosphamide i.v. and 2 mg/kg furosemide i.v. were administered, and the patient was discharged at the request of the owner. Afterwards, due to the refusal of the owner, no further treatment was given, and the owner repeated the removal of the subcutaneous air with a syringe at home. The cat was found dead at home on Day 40.

## Discussion and conclusions

In this case report, the patient had an orbital blowout pathological fracture associated with nasal lymphoma. This is a very unusual case and there are no similar reports in veterinary literature. After chemotherapy, the mass volume rapidly decreased, and air moved subcutaneously from the orbital blowout pathological fracture. In the present patient, the air had probably passed from the nasal cavity into the retro-orbital space and then in the subcutaneous space with a valve-effect on the fracture itself. Then the air in the head and neck likely move to the mediastinum through the fascial planes and there are similar cases in humans [5–7, 10].

The orbit is a pyramid-shaped cavity in the skull, a complex structure specifically responsible for visual functions [1]. The bone structure of the orbit in humans consists of frontal, ethmoidal, sphenoid, zygomatic, and lacrimal bones [11]. The orbit has four walls: a roof, a floor and the medial and lateral walls [1]. The medial wall of the orbit is composed of frontal, ethmoidal, and lacrimal bones [1], and since the floor and medial wall are the thinnest parts, they are sensitive to trauma and are often fractured [2]. Our patient showed lytic change of the medial wall (lacrimal bone) of the right orbit with a bone fragment falling off outward. The current hallmarks of human orbital blowout fracture are enophthalmos and double vision due to change of eye position, which reduces the quality of life [1]. Management for orbital blowout fracture is not required in all patients. Some patients have mild clinical symptoms or no symptoms at all [1]. Moreover, there is no criteria for surgery in the human literature [1]. Therefore, for the treatment of orbital blowout fracture, conservative or surgical treatment is applied depending on the condition of the patient. In our case, there was enophthalmos on physical examination, and it is likely that the position of the bony orbit and orbital tissue was changed to cause enophthalmos. If enophthalmos is observed in patients with nasal lymphoma, orbital fracture should be excluded.

Two main mechanisms have been suggested as the cause of orbital blowout fracture in human patients: the buckling mechanism and the hydraulic mechanism. The buckling mechanism is the theory that a traumatic force is applied to the orbital rim, and this force spreads through the bony apparatus and compresses the bone until a fracture occurs [12–17]. The hydraulic mechanism is a theory that traumatic force induces retropulsion of the eyeball, and soft tissue increases intraocular pressure to push the orbital wall outward, resulting in fracture [17–19]. It is believed that these two theories work together [20–22]. Orbital blowout fractures in humans are almost always caused by acute trauma, and non-traumatic orbital blowout fractures are very rare, but can be caused by forceful nose blowing [23, 24]. Nose blowing may induce barotrauma in the orbital rim by increasing intrasinus pressure, suggesting that blowout fracture may occur [24, 25]. However, in our case, it is most likely that a pathological fracture with secondary bone lysis caused by nasal lymphoma occurred.

In our case, since osteolysis and fracture occurred due to chronic physical compression by nasal lymphoma, surgical intervention was not performed because surgical approach was expected to be difficult and the prognosis of nasal lymphoma was expected to be short. Alternatively, conservative treatment was applied such as whole-body bandage treatment and subcutaneous drainage using a negative-pressure syringe. In both human and veterinary medicine, there is little literature on how to treat blowout fractures caused by cancers.

If subcutaneous emphysema or pneumomediastinum occurs, it is necessary to rule out other causes by checking for damage to the trachea, bronchi, or esophagus [5]. The cause of subcutaneous emphysema or pneumomediastinum is sometimes diagnosed by bronchoscopy and esophagogastroscopy in humans [5]. Human cases of subcutaneous emphysema and pneumomediastinum caused by facial fracture have been reported [5–7, 10]. Diagnosis of occult pneumomediastinum and exclusion of aerodigestive injuries are possible with thorax radiography and CT [26]. Our patient had no history of trauma. Since both of these tests were performed and an aerodigestive injury was tentatively excluded in this patient, an orbital blowout fracture could be the cause of the massive subcutaneous emphysema and pneumomediastinum. In addition, although a contrast study for esophageal perforation or bronchoscopy for tracheobronchial perforation were not performed in our patient, if these diseases were underlying conditions, our patient would most likely have symptoms such as fever, respiratory distress, and sepsis. Since these clinical symptoms were not observed, aerodigestive injuries could be tentatively excluded. In our patient, it is likely that air moved from the right nasal cavities through the frontal region and neck to the mediastinum.

In general, pneumomediastinum does not cause serious clinical problems and patients recover on their own, and most cases are clinical signs indicating an underlying condition [27]. In our case, although the cause of death was not clear because there was no follow-up, given the underlying condition, there is a possibility that the patient died due to neglect of adverse events of chemotherapy and no aggressive intervention for cutaneous emphysema and pneumomediastinum.

Although orbital blowout pathological fracture as a result of feline nasal lymphoma is a very rare complication, early diagnosis is important because the lymphoma responds immediately to chemotherapy and emphysema and pneumomediastinum can eventually occur. In nasal lymphoma, complete work-up for detecting complications associated with lymphoma should be carried out to exclude orbital blowout pathological fractures with severe complications before chemotherapy.

## Data Availability

The original contributions presented in the study are included in the article, and further inquiries can be directed to the corresponding author.

## Abbreviations

CT: Computed tomography
i.v.: Intravenous
PARR: PCR for antigen receptor rearrangement
s.c.: Subcutaneous
